# The Mediator Role of Routines on the Relationship between General Procrastination, Academic Procrastination and Perceived Importance of Sleep and Bedtime Procrastination

**DOI:** 10.3390/ijerph18157796

**Published:** 2021-07-22

**Authors:** Paula Magalhães, Beatriz Pereira, André Oliveira, David Santos, José Carlos Núñez, Pedro Rosário

**Affiliations:** 1 Psychology Research Center, School of Psychology, Campus de Gualtar, University of Minho, 4710-052 Braga, Portugal; beatriznpereira94@gmail.com (B.P.); andreoliveira@outlook.com.au (A.O.); a79890@alunos.uminho.pt (D.S.); prosario@psi.uminho.pt (P.R.); 2 Faculty of Psychology, University of Oviedo, 33003 Oviedo, Spain; jcarlosn@uniovi.es

**Keywords:** bedtime procrastination, academic procrastination, general procrastination, sleep, university students

## Abstract

Background: Sleep plays a key role in our overall function, and sleep insufficiency has been highlighted as a major health issue. ‘Bedtime procrastination’—i.e., needlessly delaying the time one goes to bed without external reasons—is one reason for sleep insufficiency. The present research aims to explore the interrelationships among Bedtime Procrastination, other domains of Procrastination, and routine-related variables. Methods: The mediating effects of Wake-up Time and Dinner Time on the relationship between Bedtime Procrastination and General Procrastination, Academic Procrastination, and Perceived Importance of Sleep were tested. Self-reported questionnaires were used, and the sample comprised of 446 university students. Results: A partial mediation model was found. General Procrastination, Academic Procrastination, and Perceived Importance of Sleep showed direct effects on Bedtime Procrastination. Moreover, Academic and General Procrastination were positively associated with Bedtime Procrastination, whereas Perceived Importance of Sleep was negatively associated with Bedtime Procrastination. Indirect effects of the Perceived Importance of Sleep and General Procrastination, as mediated by Wake-up Time and Dinner Time, on Bedtime Procrastination were also found. Conclusions: Personal routines (Wake-up Time and Dinner Time) along with individual characteristics (General and Academic Procrastination) and beliefs (perceived importance of sleep) may affect Bedtime Procrastination. Present results highlight the complexity of Bedtime Procrastination.

## 1. Introduction

Sleep plays a fundamental role in the restoration of the organism, as well as in the maintenance of homeostasis and the overall functioning of the individual [1]. Literature shows that sleep loss or short sleep duration can have a detrimental impact on the mental health (e.g., contribute to depression and anxiety symptoms, suicidal ideation) [2,3,4,5] and physical health (e.g., increased susceptibility to infectious diseases, diabetes, obesity, and cardiovascular diseases) of individuals [6,7,8,9]. Sleep insufficiency is a widespread problem that not only affects individuals with sleep-related disorders, but the general population as well [10,11]. Although causes for sleep insufficiency are manifold, one possibility is that individuals are procrastinating their bedtime, and thus not having a full night of sleep [12].

Bedtime Procrastination is the delay of sleep time before going to bed, in the absence of external reasons that justify these behaviors [12]. Bedtime Procrastination is associated with later wake-up times, later dinner times [13,14,15], morningness and eveningness [16,17], depression symptoms, anxiety, and insomnia [18,19]. Needlessly delaying sleep time has already been researched in a variety of populations and within different age groups [12,13,16,20], with literature showing that university students tend to engage in more Bedtime Procrastination compared to the general population [13].

Most procrastination arises as a means to avoid an aversive task; however, sleep is considered by most people an enjoyable task [21]. Explanations for this complex phenomenon have come forth, such as the influence of a person’s chronotype (i.e., biological approach) [16,20], i.e., eveningness and morningness, with eveningness-type adolescents showing a greater tendency to postpone bedtime; a self-regulation failure (i.e., psychological perspective), through the lack of self-control and inability to resist distractors [12,22]. Other reasons for procrastinating bedtime could be the pre-bedtime routines (e.g., flossing, taking out contacts) if they become aversive to the person [23]. Individuals can also procrastinate bedtime by losing track of time due to being absorbed in an activity, deliberately delaying it to have more time to engage in other activities, or purposely delaying it to fall asleep more easily [15].

Considering the complexity of the phenomenon of Bedtime Procrastination and from a psychological point of view, the present paper aims to contribute to the Bedtime Procrastination literature by examining the roles that some individual and routine-related factors may contribute to this behavior. Particularly, we examined the relationships of (i) procrastination in two different life domains (i.e., general and academic); (ii) the individual’s perceived importance of sleep; and (iii) routine-related variables (i.e., Dinner Time and Wake-up Time), with Bedtime Procrastination.

There is an extended scientific debate around the nature of procrastination, one commonly held view that it is a characteristic of the individual, and therefore, transversal to several life domains. The opposing understanding argues context dependency, in which case, procrastination is then domain-specific [24]. So far, research has provided support for both views. Some results suggest that procrastination is linked to genetic and personality traits, such as impulsivity [25,26], while others suggest that procrastination is a consequence of the characteristics of the task being put off, such as perceived attractiveness or difficulty [27,28]. Nevertheless, there has been a call for a more integrative view of procrastination, one that reflects a combination of individuals’ features with characteristics of the task [24].

In line with this latter proposition, we examined the role of General and Academic Procrastination on Bedtime Procrastination. Although General Procrastination as a trait is still to be fully verified, it has been associated with negative health consequences [12]. In fact, General Procrastination is associated with shorter sleep times and overall worse sleep quality [29], making it a relevant variable for our study. In addition, General Procrastination is associated with Academic Procrastination [24,27,30]. In turn, Academic Procrastination has been extensively studied over the years and is highly prevalent. In fact, it has been estimated that up to 80% of university students procrastinate at some point in their academic path [31], and inclusively, 40.5% have self-identified themselves as frequent procrastinators [32]. The detrimental impacts of procrastinating academic tasks are substantial and well documented, those of which include low academic scores, emotional distress, anxiety coupled with a feeling of failure [33,34,35], and higher amounts of stress [36]. Literature reports that for 20% to 30% of university students, procrastination presents a chronic problem with effects on academic performance [34] and quality of life [37]. Importantly, poor sleep quality and excessive sleepiness are associated with poor grades [38,39].

Regarding the variable, perceived importance of sleep, literature pertaining to the academic domain shows that attributing little importance to academic performance in a specific course is associated with high levels of procrastination on that specific course [40]. This suggests that personal views about the importance of sleep can affect Bedtime Procrastination.

Lastly, regarding the routine-related factors, we analyzed two variables that have previously been identified to be relevant in regard to Bedtime Procrastination: Dinner Time and Wake-up Time [13,14]. In a qualitative study, many people indicated that they delayed going to bed because they believed that they would be unable to sleep if they tried to bed earlier at a time when they felt excess amounts of energy [15]. We believe that this excess of energy could happen due to certain delayed routines, such as later dinner and wake-up times. In fact, previous research shows that having dinner later is associated with higher Bedtime Procrastination [14].

As stated above, the purpose of this study was to examine the effects of three predictor variables (General Procrastination, Academic Procrastination, and Perceived Importance of Sleep) on Bedtime Procrastination among university students. Particularly, we examined how this effect is mediated (totally or partially) by the routine-related variables, Dinner Time and Wake-up Time, while statistically controlling for the effects of Gender and Failed Courses, both over the mediator variables (Dinner Time and Wake-up Time) and predictor variables (General Procrastination, Academic Procrastination, and Perceived Importance of Sleep).

**Figure 1:**
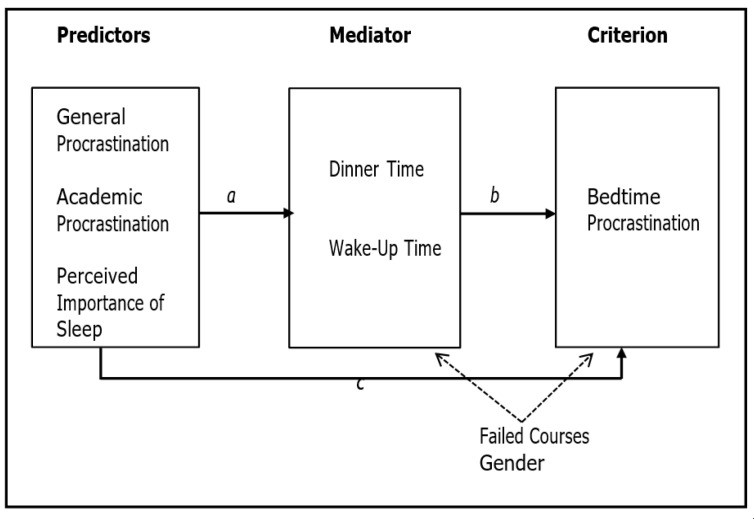
Prediction models of the Bedtime Procrastination. Total mediation model, only coefficients *a* and *b* are statistically significant; Partial mediation model, coefficients *a, b,* and *c* are statistically significant; No mediation model (only coefficient *c* is statistically significant). Failed courses and gender are treated as covariates to statistically control their effect.

Grounded on previous research, three models (Figure 1) were devised with the following hypotheses

### 1.1. Total Mediation Model

This model predicts indirect effects. It is hypothesized that the predictor variables (General Procrastination, Academic Procrastination, and Perceived Importance of Sleep) explain Bedtime Procrastination only indirectly (through Dinner Time and Wake-up Time). Particularly, it is expected that the indirect effects are positive for General and Academic Procrastination, and negative for Perceived Importance of Sleep. In Figure 1, coefficient *c* will not be statistically significant, whereas coefficients *a* and *b* will be statistically significant.

### 1.2. Partial Mediation Model

This model predicts direct and indirect effects. It is hypothesized that the predictor variables (General Procrastination, Academic Procrastination, and Perceived Importance of Sleep) explain the criterion variable (Bedtime Procrastination) both directly and indirectly through the mediator variables (Dinner Time and Wake-up Time). Particularly, it is expected that the direct and indirect effects are positive for General and Academic Procrastination, and negative for Perceived Importance of Sleep. In Figure 1, all coefficients (*a*, *b*, and *c*) will be statistically significant.

### 1.3. No Mediation Model

This model predicts only the direct effects of the predictor variables (General Procrastination, Academic Procrastination, and Perceived Importance of Sleep) over the criterion variable (Bedtime Procrastination). It is hypothesized that the three-predictor variables significantly and directly explain Bedtime Procrastination. Particularly, it is expected that both General and Academic Procrastination positively explain Bedtime Procrastination, whereas the Perceived Importance of Sleep negatively explains Bedtime Procrastination. Moreover, indirect effects of the predictor variables over the criterion variable are not expected, through either Dinner Time or Wake-up Time. In Figure 1, coefficient c will be statistically significant and positive, whereas coefficients a and b will not be statistically significant.

## 2. Materials and Methods

### 2.1. Participants

A total of 733 university students enrolled in the online survey. From this total, 287 were excluded from the analysis because they did not complete the full questionnaire (completion rate of 60.8%). Of the 446 participants that voluntarily completed the full survey, 312 (70.0%) were female. The mean age was 23.7 years (*SD* = 5.49, range 18–57), 174 (39.0%) were bachelor’s degree students, 206 (46.2%) were master’s degree students, and 66 (14.8%) were doctorate’s degree students. Participants were enrolled in distinct domains as follows: sciences and technology, social sciences, medicine, economics and management, arts and humanities, and law.

### 2.2. Instruments

#### 2.2.1. Socio-Demographic Questionnaire

The socio-demographic questionnaire included questions about the participants’ age, sex, university degree, current year of enrollment, and number of failed courses.

#### 2.2.2. Routine-Related Variables

The routine-related variables included were that of Dinner Time and Wake-up Time, assessed by asking participants about the time interval they usually have dinner (e.g., before 7:00 p.m., between 8:30 p.m. and 10:00 p.m., after 10:00 p.m.) and the time interval they usually wake-up (e.g., before 7:00 a.m., between 8:00 a.m. and 9:00 a.m.).

#### 2.2.3. General Procrastination Scale (GPS-9)

GPS-9 is a short version of Lay’s General Procrastination Scale [41] that reliably assesses global, trait-like tendencies towards procrastination across a variety of tasks [42]. This is a nine-item instrument (e.g., “I generally delay before starting work I have to do”), with three of these items being reverse scored (e.g., “I often have a task finished sooner than necessary”). All items are answered in a five-point Likert-like scale from 1 (false) to 5 (true of me). Total scores ranged between 9 and 45, with higher scores indicating a greater tendency towards general, or chronic, procrastination. The current adapted version for the Portuguese population has a Cronbach’s alpha of 0.89.

#### 2.2.4. Academic Procrastination Scale

To measure procrastination towards academic tasks, the instrument developed by Kljajic and Gaudreau [40] was used. This is a three-item instrument (e.g., “How often do you procrastinate on academic tasks in general?”) and items are answered on a five-point Likert-like scale from 1 (never) to 5 (always). Totals scores ranged from 3 to 15 with higher scores indicating more procrastination on academic tasks. The current adapted version to the Portuguese population has a Cronbach’s alpha of 0.85.

#### 2.2.5. Perceived Importance of Sleep Scale

This instrument was built for the purposes of the present study and was based on the ‘Importance scale’ developed by Kljajic & Gaudreau [40]. This is a three-item instrument (e.g., “How important is it for you to have a good night’s sleep?”) with the aim of capturing participants’ perceived value of sleep. Items were answered on a five-point Likert-like scale from 1 (not important at all) to 5 (totally important). Total scores ranged from 3 to 15, with higher scores indicating higher perceived sleep value. A factor analysis was conducted. The Kaiser–Meyer–Olkin measure verified the adequacy of the sample for the analysis with the value of KMO = 0.73. Bartlett’s test of sphericity χ2 (3) = 936.726 was significant (*p* < 0.001) and indicated that it was appropriate to apply a principal component analysis. Data showed a solution with one factor, accounting for 84.24% of the variance (Cronbach’s alpha of 0.90).

#### 2.2.6. Bedtime Procrastination Scale

The Portuguese version of the Bedtime Procrastination Scale [14], originally developed by Kroese et al. [10], was used to evaluate Bedtime Procrastination. The adapted version is a nine-item instrument (e.g., “I go to bed later than I had intended”), with four of these items being reverse scored (e.g., “I can easily stop engaging with my activities when it is time to go to bed”). All items are answered in a five-point Likert-like scale from 1 (never) to 5 (always). Total scores ranged between 9 to 45 with higher scores indicating more engagement in Bedtime Procrastination (Cronbach’s alpha for the current study = 0.90).

### 2.3. Procedure

The online survey targeting university students was shared via social media (e.g., Facebook, WhatsApp), the university institutional e-mail, and through personal contacts. Qualtrics Survey Software© ^2021^ Qualtrics^®^ (Qualtrics, Provo, UT, USA) was used as the primary tool in the construction and data collection of the survey. An informed consent was obtained from each participant prior to their participation in the study. Participation was voluntary, anonymous, and unpaid, and participants could quit the survey anytime without prejudice. Students were invited to respond individually to the self-reported questionnaires that included all the aforementioned measures. Completion of the survey took an average of 10 min. Data collection took place between December 2019 and January 2020.

### 2.4. Data Analysis

The data were analyzed as follows. First, we analyzed the statistical properties of the variables included in the study (means, standard deviations, asymmetry, and kurtosis), as well as the correlation matrix and the missing values. As the percentage of missing values was low (*n* = 21; 0.59%), they were treated through the multiple imputation procedure. Secondly, the path models of Figure 1 (no mediation, total mediation, and partial mediation) were fit with the AMOS 22 program in IBM^®^ SPSS^®^ [43]. The strategy followed for the adjustment of the three models was as follows: (i) calculate the adjustment of the three basic models (no mediation, total mediation, and partial mediation); (ii) select the best model of the three and adjust it again including the two covariates (gender and failed courses); (iii) re-specify the model eliminating statistically non-significant coefficients. The final mediational model was fitted using the bootstrapping procedure. This procedure is very useful when the sample of participants is not very large, as in our case, and its objective is to reduce bias within the analysis, and thus ensure accurate statistics, thanks to the random resampling of the sample.

The models were adjusted, and results were evaluated according to the typically used criteria: Chi-square, Root Mean Square Residual (RMR), Goodness of Fit Index (GFI), Adjusted Goodness of Fit Index (AGFI), Tucker-Lewis Coefficient (TLI), Comparative Fit Index (CFI), Root Mean Square Error of Approximation (RMSEA), Akaike Information Criterion (AIC), Bayes Information Criterion (BIC), and Expected Cross-Validation Index (ECVI). While the first seven indexes provide information on the degree of fit of the theoretical model to the collected data, AIC and BIC are used to decide which competing model best fits. Finally, ECVI informs us of the extent to which these results could be replicated in an independent sample. There is evidence of a good fit when χ^2^ has a *p* > 0.05; RMR < 0.05; GFI, AGFI and TLI ≥ 0.90; CFI ≥ 0.95; and RMSEA ≤ 0.06. The best model is the one that gets the smallest AIC and/or BIC. On the other hand, there is evidence of safety in the data obtained when the ECVI of our model is lower than that of the saturated model. The effect size of the regression coefficients was evaluated using Cohen’s *d* statistic [44].

## 3. Results

### 3.1. Descriptive Statistics

Table 1 shows the descriptive statistics and correlation matrix of the variables included in the model. Results show that 67.8% of the correlations are statistically significant and the Bartlett sphericity test indicates that the correlation matrix is adequate for the analysis (χ^2^ (28) = 560.34, *p* < 0.001). Moreover, the Kaiser–Meyer–Olkin for the sampling adjustment is acceptable (KMO = 0.649). Additionally, regarding the criteria established by Gravetter and Walnau [45], asymmetry and kurtosis indicate a normal, univariate, distribution of the data, except for the variable, Perceived Importance of Sleep. Altogether, the Mardia coefficient suggests a multivariate normality of the data (*M* = 1.685; *t* = 1.407; *p* > 0.05).

Correlational tests indicate no statistically significant difference between females and males regarding the three types of procrastination (General, Academic, and Bedtime). However, females show a tendency to ascribe more value to sleep (Perceived Importance of Sleep) than males. In addition, females have less failed courses than males. Finally, the three procrastination variables are highly, positively associated with each other, and significantly positively correlated with Dinner Time and Wake-up Time.

### 3.2. Selection of the Best Model

The three aforementioned models were tested: no mediation model (Model 1 in Table 2), total mediation model (Model 2 in Table 2), and partial mediation model (Model 3 in Table 2). Considering the criteria previously described (i.e., Chi-square, RMR, GFI, AGFI, TLI, CFI, RMSEA), results of the goodness of fit of the models indicate the rejection of the total mediation model (Model 2) (all indices indicate a poor adjustment). Similarly, the no mediation model (Model 1) shows some level of adjustment although not completely satisfactory (see Table 2). The RMR, GFI, and the AGFI scores allow for the acceptance of Model 1, still, other parameters, such as TLI or CFI, and especially RMSEA, suggest rejection of the model. Lastly, Model 3, which includes direct and indirect effects (partial mediation model), shows a good fit. However, this model is unstable since it has zero degrees of freedom, which means that no probability level can be assigned to the chi-square statistic. Thus, we added some free parameters, i.e., those parameters that were not statistically significant. Consequently, the result of the fit of the partial mediation model re-specified (Model 3-R in Table 2) was completely satisfactory. Lastly, Model 3-R was fit by adding gender and failed courses as co-variables (Model 3-R’ in Table 2). Results indicate a good fit of the model, although not as good as the fit of Model 3-R without the co-variables.

To summarize, the goodness-of-fit indexes suggest that the model including direct and indirect effects re-specified (partial mediation model), without co-variables, is the best fit among the three models tested. Additionally, statistics of the AIC and BIC of this model are the smallest of the three models (AIC = 37.929; BIC = 111.735). Therefore, all data converge to select Model 3-R as the best fit. Lastly, Model 3-R is the only one in which the ECVI value is lower than that of the saturated model (default model = 0.085 vs. saturated model = 0.094). This finding suggests that this model is reliable to predict future models, with good fit in independent samples of similar size.

### 3.3. Direct, Indirect, and Total Effects in the Bedtime Procrastination Model (Model 3-R)

Table 3 shows the standardized regression coefficients of the direct and indirect effects, as well as the co-variances, confidence intervals at 90%, estimation errors, significances for the estimated coefficients, and the effect size for the fit of Model 3-R (partial mediation model).

**Figure 2:**
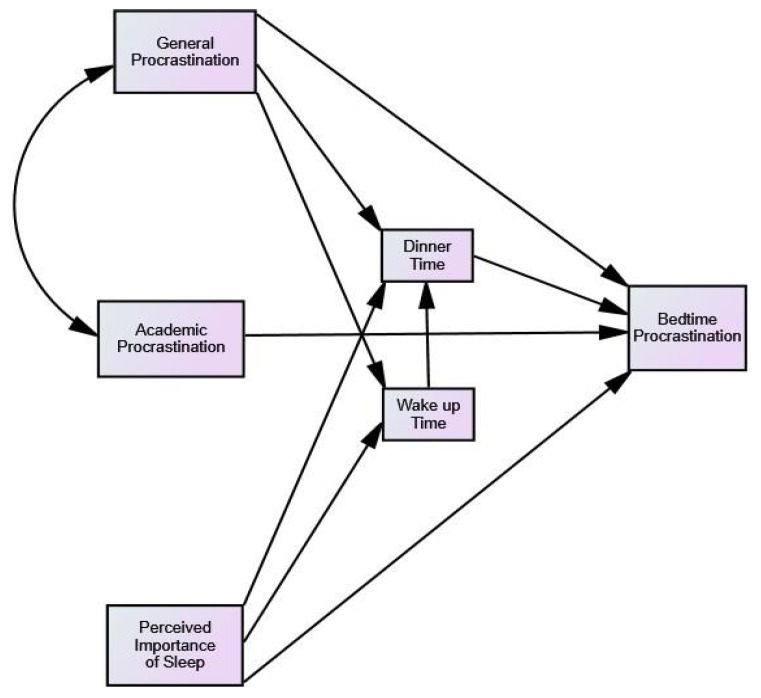
Direct effects on the Bedtime Procrastination Model.

Data support the partial mediation model of Dinner Time and Wake-up Time on the relationship between General Procrastination, Academic Procrastination, and Perceived Importance of Sleep and Bedtime Procrastination. Note that the inclusion of the co-variables, Gender and Failed Courses, in the model did not result in a better fit of the model. Figure 2 displays the direct effects that were statistically significant. First, the three predictor variables (General Procrastination, Academic Procrastination, and Perceived Importance of Sleep) directly explain the criterion variable (Bedtime Procrastination). Likewise, General Procrastination and Perceived Importance of Sleep also explain Bedtime Procrastination indirectly through the mediator variables, Dinner Time and Wake-up Time. Contrary to our hypothesis, Academic Procrastination only has a direct effect over Bedtime Procrastination. Overall, data indicate that the greater the General and Academic Procrastination and the less the Perceived Importance of Sleep, the greater the Bedtime Procrastination.

Regarding the indirect effects, data indicate that the total indirect effects are smaller than expected, i.e., smaller than the direct effects. Dinner Time and Wake-up Time are only marginally mediator variables (the size of the indirect effects is not excessively relevant). Furthermore, regarding the independent variables of the model (General Procrastination, Academic Procrastination, and Perceived Importance of Sleep), only the procrastination variables are related with each other. Lastly, it is important to highlight that the criterion variable of the model (Bedtime Procrastination) is significantly explained by the model, despite the modest amount of variance explained (25%).

## 4. Discussion

Sleeping the recommended number of hours is vital for various brain functions [11,46], learning [47,48], and maintenance of physical and mental health [48,49]. Bedtime Procrastination is one reason that may inhibit an individual from attaining this amount of recommended hours, becoming an obstacle to overall individual functioning [10]. Hence, to overcome sleep insufficiency, it seems important to deepen the knowledge about the Bedtime Procrastination phenomenon.

Literature has been stressing the importance of considering the transversal nature of procrastination across different domains of an individual’s life [24]. Thus, in this study, we examined procrastination in three different domains: General, Academic, and Bedtime. In addition, regarding Bedtime Procrastination, it has been suggested that routines may play a key role in this behavior, although data on this topic is limited [13,14,15,24]. Thus, this study also attempts to add to literature on this aspect. All considered, the purpose of this paper was to explore the interrelationships among Bedtime Procrastination, Academic Procrastination, General Procrastination, Wake-up Time, Dinner Time, and Perceived Importance of Sleep. Additionally, the mediational role of Wake-up Time and Dinner Time on the relationship of General Procrastination, Academic Procrastination, and Perceived Importance of Sleep, with Bedtime Procrastination was also explored.

Results showed that there is a relationship between General Procrastination and Academic Procrastination; particularly being that the higher the procrastination towards general tasks (e.g., shopping at the last minute), the higher the procrastination in academic tasks (e.g., delay academic tasks beyond the reasonable). This result is consistent with previous literature on Academic Procrastination that found a correlation with General Procrastination [24,27,30]. Moreover, in the present study, both General Procrastination and Academic Procrastination were positively associated with, and provide explanation for, Bedtime Procrastination. The relationship between these variables suggests that individuals who are more prone to procrastinate in general (e.g., daily life tasks) and academically (e.g., finishing a paper), are also more likely to delay their intended hour of going to bed. Although there is a lack of research on this topic, Li et al. [29] found that General Procrastination was indeed associated with shorter sleep times and overall worse sleep quality, which according to our results, could be the result of Bedtime Procrastination. Present data could also help to explain the relationship between poor sleep quality and excessive sleepiness with poor grades [38,39]. The direct effects of the present model contribute to the idea that procrastination may be transversal to different life domains [26,27,37,50,51]—i.e., an individual who procrastinates in one life domain is more likely to procrastinate in other domains.

Still, regarding the direct effects of the present model, Perceived Importance of Sleep was negatively associated with Bedtime Procrastination, i.e., individuals who attribute less value to sleep were more likely to procrastinate when it was time to go to bed. This result is similar to previous findings regarding Academic Procrastination, where little importance being attributed to performing well in a certain course is associated with high levels of procrastination on that specific course [40]. This suggests that changes on personal views about the importance of sleep could also bring about changes upon Bedtime Procrastination.

Lastly, regarding the indirect effects of the model tested, a mediational role of Dinner Time on the relationship between General Procrastination and Perceived Importance of Sleep and Bedtime Procrastination was found. That is, the more students procrastinate in general tasks, and the less they perceive sleep as important, the later they have dinner; and the later they have dinner, the more they procrastinate their bedtime. This means that the effect of General Procrastination and Perceived Importance of Sleep on Bedtime Procrastination is likely to occur when students have dinner later in the evening. Magalhães et al. [14] also found that Bedtime Procrastination was associated with later dinner times. In addition, although students who procrastinate in general tasks and attribute less value to sleep are more likely to wake-up later, Wake-up Time does not predict Bedtime Procrastination. Nevertheless, individuals who wake-up later tend to have dinner later too. Altogether, these results suggest that a lack of routine may contribute to Bedtime Procrastination.

Overall, the present study confirmed the partial mediation model hypothesis, i.e., the predictor variables (General Procrastination, Academic Procrastination, and Perceived Importance of Sleep) explain Bedtime Procrastination, both directly and indirectly, through one mediator variable (Dinner Time). In fact, there is an effect of the predictor variables on dinnertime, and there is an effect of Dinner Time on Bedtime Procrastination. However, even if participants have dinner earlier, they are still likely to procrastinate their bedtime if they procrastinate in general tasks and perceive sleep to have little importance.

All considered, the present results contribute to a continuously expanding view that procrastination could be transversal to several life domains of the individual [24]. Additionally, these results also suggest that accomplishing routines may be important to curbing Bedtime Procrastination. Thus, the plan for mitigating Bedtime Procrastination should include the promotion of sleep routines, as well as the promotion of strategies that support routine schedules in different life domains at the same time. To address these aspects, the design of future interventions may wish to focus on training self-regulation strategies related to routines and time management that could be applied to different domains of life. For example, design programs that address priority-setting skills (e.g., start a new activity or prepare to go to bed) and monitoring skills (e.g., reminders that it is almost time for dinner) [15]. Moreover, programs could also consider including an initial session with a brief intervention focusing on the relevance and benefits of sleep to improve the individual’s Perceived Importance of Sleep. This session could include information about the importance of sleep for health and overall functioning. In fact, there is evidence that brief interventions are cost-effective in promoting positive attitudes towards different topics [52,53]. Particularly, research in the academic domain shows that interventions focusing on highlighting the relevance of a topic are effective in improving the utility value that students attribute to that topic [54]. Thus, this same strategy could be effective regarding the sleep domain.

Although the present research entails an advancement in the understanding of the Bedtime Procrastination phenomenon, some potential limitations should be acknowledged when interpreting the results. First, the study is correlational, which does not provide solid grounds for causality of the effects. Future research could use a longitudinal design to understand how stable these behaviors may be throughout time, and if changes in one variable could lead to changes in others. Second, the data collection was entirely conducted online through self-reported questionnaires. Nevertheless, since the measures used reported a high reliability, we believe that this limitation did not affect our results. Moreover, it is not possible to guarantee the representativeness of the present findings, since we did not ask the participants about their area of residence. Nevertheless, the current study’s survey was shared with students from different universities from several areas of Portugal (e.g., North, South, Islands), which gives us confidence that a wide range of socioeconomic and cultural backgrounds have been covered. In addition, it was not possible to guarantee a balance between genders of the respondents. Lastly, the present study considers variables that add to the literature, suggesting that procrastination intersects several life domains of the individual. However, studies integrating several domains of procrastination are scarce, which highlights the need to further examine the transversal nature of the phenomenon, while continuing to explore domain-specific aspects. In the present study, we looked at the issue of Bedtime Procrastination from a psychological point of view. However, as suggested by Kroese et al. [55], we recognize that the phenomenon of Bedtime Procrastination is complex, and can be researched from different perspectives (e.g., biological). Therefore, future research could consider studying the role of distinct types of variables or aspects that may be unique to Bedtime Procrastination, such as the bedroom environment, household sleep and meal routines, tiredness, and circadian preferences (i.e., morningness/eveningness). Particularly, considering that research has been emphasizing the role of light on alertness and sleep [56] as well as the influence of a person’s chronotype on Bedtime Procrastination [20], the design of future studies could include physiology-based circadian model measures as controls of Bedtime Procrastination.

## 5. Conclusions

Overall, the results of the present study contribute to the existing literature, shedding light on the continuously expanding view that procrastination could be transversal to several life domains of the individual. To understand more deeply the etiology of Bedtime Procrastination, we tested the mediating and indirect roles of Wake-up Time and Dinnertime on the relationship between Academic Procrastination and General Procrastination and Bedtime Procrastination, as well as the subjective value attributed to sleep. Our results showed that the predictor variables explain Bedtime Procrastination both directly and indirectly, through dinnertime. These findings are expected to shed some light in the design of future interventions on Bedtime Procrastination. For example, the promotion of strategies that help to accomplish routines (e.g., dinnertime) may be important in reducing Bedtime Procrastination. Finally, future research could be developed with a longitudinal design and consider the study of other possible predictors that may have a significant influence on Bedtime Procrastination. For example, perception of tiredness throughout the day and the role of circadian rhythm of the individuals.

## Figures and Tables

**Table 1:** Descriptive statistics and Pearson correlations.

−	GP	AP	PIS	BP	DT	WU	FC	GE
GP	−							
AP	0.714 **	−						
PIS	−0.060	0.021	−					
BP	0.429 **	0.388 **	−0.242 **	−				
DT	0.161 **	0.119 *	−0.171 **	0.195 **	−			
WU	0.178 **	0.145 **	−0.113 *	0.161 **	0.202 **	−		
FC	0.150 **	0.159 **	−0.043	0.036	0.049	0.124 **	−	
GE	−0.073	−0.055	0.149 **	−0.050	−0.013	−0.222 **	−0.094 *	−
M	3.122	3.314	4.624	3.234	2.560	2.520	0.314	1.700
SD	0.818	0.883	0.602	0.885	0.610	0.865	0.465	0.459
SK	−0.114	−0.130	−1.813	−0.341	0.176	0.261	0.805	−0.873
KU	−0.417	−0.436	3.467	−0.756	−0.401	−0.682	−1.359	−1.234

General procrastination (GP); Academic procrastination (AP); Perceived importance of sleep (PIS); Bedtime Procrastination (BP); Dinner Time (DT); Wake-up Time (WU); Failed courses (FC); Gender (GE; 1 = boys, 2 = girls); Mean (M); Standard deviation (SD); Skewness (SK); Kurtosis (KU).* *p* < 0.05; ** *p* < 0.01.

**Table 2:** Selecting the best of competing models.

	Direct Effects Model (No Mediation) Model 1	Indirect Effects Model (Total Mediation) Model 2	Direct and Indirect Effects Model (Partial Mediation-R) Model 3-R	Partial Mediation Model-R (with Gender and Failed Courses as Covariables) Model 3-R’
χ^2^	37.248	109.313	1.929	33.403
*df*	6	3	3	14
*p*	<0.001	<0.001	0.587	0.003
RMR	0.051	0.087	0.009	0.021
GFI	0.974	0.932	0.999	0.981
AGFI	0.909	0.526	0.990	0.952
TLI	0.843	0.070	0.968	0.928
CFI	0.937	0.786	0.987	0.964
RMSEA	0.108	0.282	0.032	0.056
AIC	67.248	145.313	37.929	77.403
BIC	128.753	219.119	111.735	167.610
ECVI				
Default model	0.151	0.327	0.085	0.174
Saturated model	0.094	0.094	0.094	0.162

**Table 3:** Results of the fit of Model 3-R (partial mediation model) with bootstrapping procedure (500 bootstrap samples).

	Estimate	LO90	UP90	SE	*p*	*d*
**Direct Effects**						
AP → BP	0.195	0.079	0.316	0.071	<0.001	0.319
GP → WU	0.172	0.097	0.240	0.045	<0.001	0.355
GP → DT	0.151	0.049	0.200	0.046	0.006	0.253
GP → BP	0.276	0.165	0.387	0.066	<0.001	0.429
PIS → WU	−0.102	−0.187	−0.025	0.049	0.026	0.209
PIS → DT	−0.162	−0.221	−0.055	0.050	0.018	0.303
PIS → BP	−0.229	−0.278	−0.152	0.038	<0.001	0.502
WU → DT	0.164	0.084	0.236	0.047	<0.001	0.338
DT → BP	0.093	0.020	0.163	0.043	0.030	0.212
**Total Indirect Effects**						
GP → BP via DT	0.014	0.005	0.031	0.007	0.067	0.174
PIS → BP via DT	−0.015	−0.035	−0.004	0.009	0.076	0.169
WU → BP via DT	0.015	0.004	0.033	0.008	0.071	0.171
**Covariances**						
AP → AG	0.515	0.450	0.575	0.038	<0.001	1.425
AP → PIS	0.011	−0.026	0.053	0.024	0.645	0.043
PIS → AG	−0.030	−0.062	0.011	0.022	0.187	0.120

Estimate (standardized regression weights), LO90 (Lower 90%), UP90 (Upper 90%), SE (standardized errors), General procrastination (GP); Academic procrastination (AP); Perceived importance of sleep (PIS); Bedtime Procrastination (BP); Dinner Time (DT); Wake-up Time (WU).

## Data Availability

Data is available from the first author upon reasonable request.
